# Characterization of the complete chloroplast genome of *Delphinium grandiflorum* L.

**DOI:** 10.1080/23802359.2019.1692707

**Published:** 2019-12-09

**Authors:** Huirong Duan, Yuan Lu, Xiaorong Duan, Xuehui Zhou, Chunmei Wang, Fuping Tian, Xiaoli Wang, Hongshan Yang, Guangxin Cui

**Affiliations:** a Lanzhou Institute of Husbandry and Pharmaceutical Science, Chinese Academy of Agricultural Sciences, Lanzhou, Gansu, China;; b Shanxi Electric Power Research Institute, State Grid Corporation of China, Taiyuan, Shanxi, China

**Keywords:** *Delphinium grandiflorum* L., chloroplast genome, phylogenetic relationship

## Abstract

*Delphinium grandiflorum* L. is a perennial herb, and has very high medicinal value. However, the evolutionary relationship analysis of *D. grandiflorum* is limited. Its cp genome was 157,339 bp in length, containing a pair of inverted repeated regions (52,304 bp), separated by a large single copy region of 88,098 bp, and a small single copy region of 16,937 bp. Moreover, a total of 117 functional genes were annotated, including 79 mRNA, 30 tRNA genes, and 8 rRNA genes. The phylogenetic relationships inferred that *D. grandiflorum* was closely related to *Gymnaconitum gymnandrum*. This study will provide a theoretical basis for species identification and biological research.

*Delphinium grandiflorum* L., belonging to the family Ranunculaceae, is a perennial herb mainly distributed in some regions of Siberia, the Northwest of China, and People’s Republic of Mongolia (Chen et al. 2017). *Delphinium grandiflorum* can be used as a folk medicine for the treatment of toothache and native pesticide (Zhang et al. 2012). However, the evolutionary relationship of *D. grandiflorum* is limited in study. Here, the complete cp genome of *D. grandiflorum* was sequenced on the Illumina NovaSeq platform (Benagen Tech Solution Co., Ltd, Wuhan, China), which will provide a theoretical basis for species identification and biological research.

The fresh leaves of *D. grandiflorum* were collected in Lanzhou Scientific Observation and Experiment Field Station of the Ministry of Agriculture for Ecological System in the Loess Plateau Area (36lecte, 103lected in Lanzho1700 m), Gansu, China, on 22 July 2019. The voucher specimen was kept in Herbarium of Lanzhou Institute of Husbandry and Pharmaceutical Science, Chinese Academy of Agricultural Sciences (CYSLS-DgDUAN20190722). The total genomic DNA of *D. grandiflorum* was extracted from the fresh leaves with a modified CTAB method (Li et al. 2018). One library (250 bp) was constructed using pure DNA according to the manufacturer’s instructions (NEBNext^®^Ultra^TM^ DNA Library Prep Kit for Illumina^®^). The library was performed with an Illumina NovaSeq platform (Benagen Tech Solution Co., Ltd, Wuhan, China) and 150 bp paired-end reads were generated. Approximately 6.4 GB of clean data were yielded. The assembled reads were joined into bidirectional iterative derivation using NOVOPlasty (version: 32, parameter: k-mer = 39) to obtain the whole-genome sequence (Dierckxsens et al. 2017). The assembled genome was annotated using GeSeq (Tillich et al. 2017). The circular gene map of *D. grandiflorum* was drawn using the OGDRAWv1.2 program (Loshe et al. 2007).

The complete cp genome of *D. grandiflorum* is 157,339 bp in length with a typical quadripartite structure, containing a pair of inverted repeated (IR) regions (52,304 bp) that are separated by a large single copy (LSC) region of 88,098 bp, and a small single copy (SSC) region of 16,937 bp. The GC content of the whole cp genome was 38.08%. A total of 117 functional genes were annotated, including 79 protein-coding genes (mRNA), 30 tRNA genes, and 8 rRNA genes. The protein-coding genes, tRNA genes, and rRNA genes account for 67.52, 25.64, and 6.84% of all annotated genes, respectively.

The phylogenetic tree was generated based on the complete cp genome of *D. grandiflorum* and other 24 species (Figure 1). The alignment was conducted using MAFFT (Katoh and Standley 2013). The phylogenetic tree was built using the neighbor-joining (NJ) method. The results showed that *D. grandiflorum* was closely related to *Gymnaconitum gymnandrum*.

**Figure 1.**
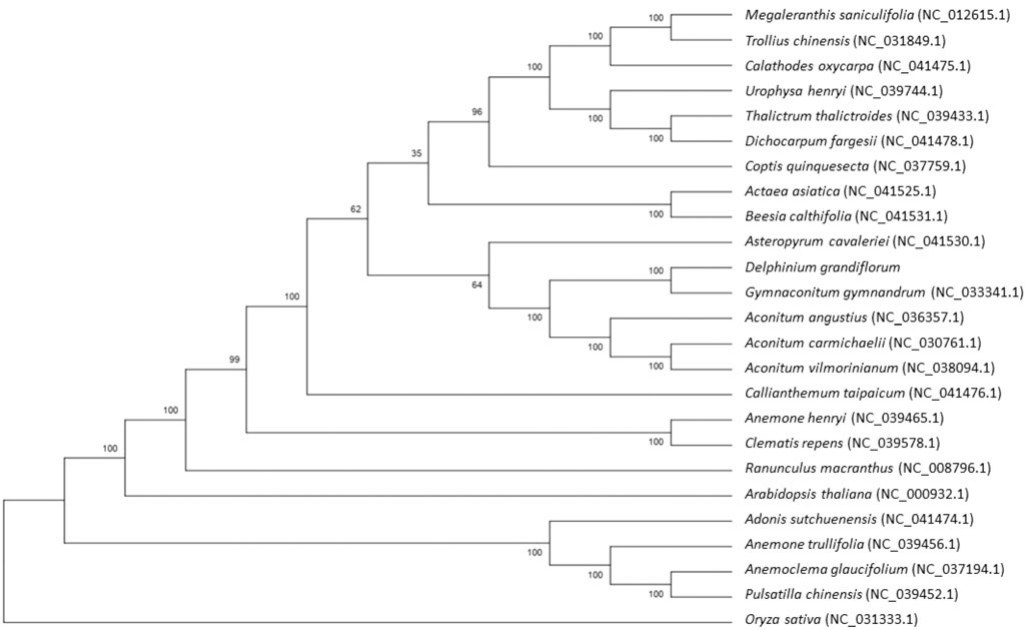
Phylogenetic relationships of 25 species based on complete chloroplast genome using the neighbor-joining methods. The bootstrap values were based on 1000 replicates and are shown next to the branches.

## References

[CIT0001] Chen NH, Zhang YB, Li W, Li P, Chen LF, Li YL, Li GQ, Wang GC. 2017. Grandiflodines A and B, two novel diterpenoid alkaloids from *Delphinium grandiflorum*. RSC Adv. 7(39):24129–24132.

[CIT0002] Dierckxsens N, Mardulyn P, Smits G. 2017. NOVOPlasty: *de novo* assembly of organelle genomes from whole genome data. Nucleic Acids Res. 45(4):e18. 28204566 10.1093/nar/gkw955 PMC5389512

[CIT0003] Katoh K, Standley DM. 2013. MAFFT multiple sequence alignment software version 7: improvements in performance and usability. Mol Biol Evol. 30(4):772–780. 23329690 10.1093/molbev/mst010 PMC3603318

[CIT0004] Li X, Li YF, Zang MY, Li MZ, Fang YM. 2018. Complete chloroplast genome sequence and phylogenetic analysis of *Quercusacutissima*. IJMS. 19(8):2443–2459. 10.3390/ijms19082443 PMC6121628 30126202

[CIT0005] Loshe M, Drechesel O, Bock R. 2007. OrganellarGenomeDraw(ORDRAW): a tool for easy generation of high quality customgraphical maps of plastid and mitochondrial genomes. Curr Genet. 52:15655. 10.1007/s00294-007-0161-y 17957369

[CIT0006] Tillich M, Lehwark P, Pellizzer T, Ulbricht-Jones ES, Fischer A, Bock R, Greiner S. 2017. GeSeq - versatile and accurate annotation of organelle genomes. Nucleic Acids Res. 45(W1):W6–W11. 28486635 10.1093/nar/gkx391 PMC5570176

[CIT0007] Zhang YN, Li B, He M. 2012. Effects of saline and alkali stress on seed germination of *Delphinium grandiflorum*. Pratacult Sci. 29:1235–1239. Chinese.

